# The impact of coronavirus disease (COVID-19) pandemic on developing obsessive-compulsive disorder in saudi arabia

**DOI:** 10.1192/j.eurpsy.2021.762

**Published:** 2021-08-13

**Authors:** D. Alateeq, H. Almughera, T. Almughera, R. Alfedeah, T. Naser, K. Alaraj

**Affiliations:** 1 College Of Medicine, Princess Nourah bint Abdulrahman University, Riyadh, Saudi Arabia; 2 College Of Medicine, Princess Noura Bint Abdulrahman university, Riyadh, Saudi Arabia; 3 College Of Medicine, Princess noura university, Riyadh, Saudi Arabia; 4 College Of Medicine, Princess Noura university, Riyadh, Saudi Arabia; 5 Collage Of Medicine, Princess nourah university, Riyadh, Saudi Arabia

**Keywords:** Obsessive-Compulsive disorder, Coronavirus Disease, ocd, COVID-19

## Abstract

**Introduction:**

Coronavirus disease (COVID-19) is a contagious disease. Its potential psychological impact could involve fear of being contaminated by germs and dirt, which may lead to washing hands repeatedly until harm the skin.

**Objectives:**

To explore the incidence of Obsessive-Compulsive Disorder (OCD) symptoms during COVID-19 pandemic among the Saudi general population, and to explore its correlation with stress and the associated factors.

**Methods:**

A cross-sectional survey of a sample consisting of 2909 participants was conducted during COVID-19 outbreak consists of socio-demographic characteristics, Perceived Stress Scale (PSS) and The Brief Obsessive–Compulsive Scale (BOCS).

**Results:**

Most participants were female (73.9%) with university level or above (81%) and were disciplined with quarantine (75.6%). New onset symptoms of obsessive thoughts (worries about germs, dirt and viruses), and compulsive behavior (excessive hand washing) were reported by 57.8% and 45.9% of the participant. Participants who developed these symptoms only during CODIV-19 pandemic were significantly higher than asymptomatic participants or those who developed symptoms before the pandemic (p-value&lt; 0.000). New onset symptoms were significantly more among participants with high stress (57.5% and 51.4%; p-value &lt;0.000). Some sociodemographic characteristics were significantly associated with new onset OCD symptoms such as age group (40-49 years), employee in non-medical field, housewives, students, being disciplined and spending more days in quarantine (p- value&lt;0.000, p-value&lt;0.047, p-value&lt;0.012, p-value&lt;0.015).

**Conclusions:**

This study revealed a significantly higher prevalence of high perceived stress in respondents with new onset OCD symptoms. This implies that bio disaster is associated with a high psychological morbidity which needs interventional programs.

